# The association of socio-economic and psychological factors with limitations in day-to-day activity over 7 years in newly diagnosed osteoarthritis patients

**DOI:** 10.1038/s41598-022-04781-3

**Published:** 2022-01-18

**Authors:** Afroditi Kouraki, Tobias Bast, Eamonn Ferguson, Ana M. Valdes

**Affiliations:** 1 School of Medicine, University of Nottingham, Nottingham, UK; 2 School of Psychology, University of Nottingham, University Park, Nottingham, UK; 3 Pain Centre Versus Arthritis, Academic Rheumatology, City Hospital, Nottingham, UK; 4 NIHR Nottingham Biomedical Research Centre, University of Nottingham, Nottingham, UK; 5 Neuroscience@Nottingham, University of Nottingham, Nottingham, UK

**Keywords:** Epidemiology, Musculoskeletal system

## Abstract

Previous research has established links between chronic pain and impaired cognitive ability, as well as between chronic pain and anxiety, in osteoarthritis. Furthermore, there is evidence linking risk of osteoarthritis to lower educational attainment. However, the inter-play of these factors with key social factors (e.g., social deprivation) at the early stages of osteoarthritis are not understood. Here, we used data from waves 4, 5, 6 and 7 of the Survey of Health, Ageing and Retirement in Europe (SHARE) (n = 971) and selected a subsample of respondents who initially did not report a diagnosis of osteoarthritis until wave 6. We used path models to test how social deprivation, education and anxiety, before diagnosis (waves 4 and 5), affect the relationship between cognitive ability, pain and limitations in activities of daily living following diagnosis (waves 6 and 7). We show that high social deprivation before diagnosis predicts greater limitations in activities of daily living after diagnosis, with this effect partly mediated by impaired cognitive ability. We also find that higher educational attainment before diagnosis may protect against limitations in activities of daily living after diagnosis via better cognitive ability and lower anxiety. Therefore, improving cognitive ability and managing anxiety may mitigate the associations of social deprivation and low educational attainment with limitations in activities of daily living.

## Introduction

Osteoarthritis is the most common form of arthritis^1^ and is characterized by pain, which can lead to limitations in activities of daily living and reduced quality of life^2,3^. Indeed, cross-sectional studies report a significant association between pain severity and activities of daily living in older adults with osteoarthritis^4,5^.

Pain status in older adults with osteoarthritis is an independent predictor of limitations in instrumental activities of daily living (IADL)^6^. IADL reflect the ability to perform activities, such as doing housework, shopping, taking medications, preparing a hot meal, making calls, and managing money, independently^7^. Limitations in IADL generally occur before limitations in basic activities of daily living^8^. In older adults, they progress cumulatively^9^, and therefore interventions for preventing limitations in IADL during the early stages of osteoarthritis may limit further decline.

IADL depend on cognitive skills, mood and motivational factors, all of them negatively associated with pain^10,11^. For example, anxiety is associated with increased limitations in IADL independently of depression comorbidity^12^. In osteoarthritis, higher levels of anxiety are associated with heightened pain, as well as limitations in activities of daily living^4,13–16^. Moreover, the existing literature shows that in osteoarthritis, good cognitive ability is protective against experiencing higher pain intensity, and higher pain intensity is associated with cognitive impairment^17–20^.

Social factors may affect limitations in activities of daily living, although their role in osteoarthritis is not well understood. Indeed, individual and neighbourhood social factors have been associated with risk for developing pain that interferes with daily activities^21–24^. Furthermore, a cross-sectional link between higher social deprivation, assessed using 9-digit ZIP codes, and greater limitations in activities of daily living in musculoskeletal conditions has been reported^22^. For this study, social deprivation is defined as social isolation (i.e. feeling left out of things), lack of social support, poor quality of the local area (i.e. cleanness, vandalism, presence of helpful people, feeling part), ease of access to local amenities (e.g., the nearest bank, pharmacy, and grocery shop), and lack of social activities^25^. Limitations in activities of daily living among older people are the result not only of health problems, but also of their interactions with psychological and socioeconomic factors^26,27^. Indeed, it has also been shown that social engagement promotes better executive functioning in old age^28–31^. Thus, cognitive ability is a potential mediating mechanism in the relationship between social deprivation and limitations in IADL^11^.

In osteoarthritis, low educational attainment is associated with greater reported pain and poorer health status outcomes independently of socioeconomic factors, such as income^23,24,32–36^. There is also evidence that educational attainment has positive effects on late-life cognitive ability^37^. Therefore, higher educational attainment may protect against increased pain levels and limitations in IADL, via its association with improved cognitive ability.

It should be noted that although cognitive ability and education are correlated, they are distinct from one another, and one can act as a moderator of the other with respect to life outcomes^38^. First, there are other, especially social, determinants of whether or not people achieve certain educational outcomes^39^. Second, educational attainment is more closely related to crystallized abilities (i.e. stored knowledge that has been accumulated through learning, such as grammar and academic knowledge), than to fluid abilities, such as processing speed and abstract reasoning^40,41^. The measure of cognitive ability used in this study mainly reflects fluid cognition.

All of the factors discussed above will contribute to limitations in activities of daily living in general, but how they interact to contribute to limitations in IADL caused specifically by osteoarthritis pain is not yet well understood. Therefore, the present study builds on this previous work by exploring in more detail how social deprivation and educational attainment influence the dynamic relationship between IADL, pain, anxiety and cognitive ability before and after a diagnosis of osteoarthritis. As affective and cognitive factors are influenced by the experience of pain^17–20^, it is necessary to assess these factors prior to the emergence of disease associated with pain (osteoarthritis) in order to show their unconfounded effects on subsequent pain and limitations in IADL. This would allow us to identify factors that could be targeted for early interventions to reduce the disability caused by osteoarthritis^42^.

In the present study, we analysed data over four consecutive waves from the Survey of Health, Ageing and Retirement in Europe (SHARE), a large longitudinal survey covering 28 European countries^43^, which allowed us to follow the trajectory and development of osteoarthritis over time (i.e. before and after diagnosis). We assessed the role of social deprivation, anxiety and cognitive ability before a diagnosis of osteoarthritis in limitations in IADL, pain and cognitive ability after the onset of osteoarthritis in a selected sample of newly diagnosed osteoarthritis patients. Specifically, we tested the following hypotheses: (1) higher levels of pain will be related to impaired cognitive ability and higher anxiety after controlling for pain before diagnosis and confounders, (2) better cognitive ability will be related to reduced pain levels and lower social deprivation after controlling for cognitive ability before diagnosis and confounders, (3) greater limitations in IADL will be associated with higher anxiety and poorer cognitive ability after controlling for limitations in IADL before diagnosis and confounders and (4) pain, cognitive ability and anxiety will act as the mechanisms indirectly linking socio-economic factors (social deprivation and educational attainment) to limitations in IADL.

## Results

### Descriptives

Descriptive statistics for the sample are reported in Table 1. All fit indices suggested the model fits these data well [Root Mean Square Error of Approximation (RMSEA) = 0.044, 95% CI 0.033–0.055, Standardised Root Mean Square Residual (SRMR) = 0.026, Comparative Fit Index (CFI) = 0.983, Tucker Lewis Index (TLI) = 0.957] (see Supplementary Table S1).

**Table 1:** Descriptive statistics (n = 971).

Characteristics	W4, 2011	W5, 2013	W6, 2015	W7, 2017
**Age (53–101)**	67.04 (9.33)	69.04(9.33)	71.04(9.33)	73.04 (9.33)
**BMI (16–53)**	27.10 (6.90)	27.23 (6.77)	26.95 (7.31)	26.73 (7.51)
**No. of chronic diseases (0–9)**	2.18 (1.6)	1.97 (1.53)	3.16 (1.72)	3.27 (1.78)
**Social deprivation index**^a^ ** (0–0.65)**	–	0.20 (0.15)	–	–
**Cognitive ability total score (0–107)**	33.77 (10.82)	33.59 (11.81)	33.37 (11.46)	*
**Level of pain (1–4)**	**	2.48 (1.09)	3.09 (0.67)	*
**Anxiety**^a^ **(0–20)**	–	7.95 (2.96)	–	–
**IADL (0–7)**	0.31 (0.83)	0.39 (0.96)	0.56 (1.15)	0.72 (1.43)
**Female Gender, n (%)**	73.53	73.53	73.53	73.53
**Education, n (%)**				
Low—ISCED code 0,1 and 2	43.56	41.00	40.88	41.30
Medium—ISCED code 3 and 4	35.53	34.81	34.91	39.34
High—ISCED code 5 and 6	19.15	18.33	18.33	18.54
Other	0.62	0.62	0.61	0.62
**Marital status, n (%)**				
Married and living together	46.76	50.00	45.07	*
Divorced or widowed	21.52	45.00	49.29	*
Other	5.35	5.00	11.27	*
**Physical activity, n (%)**				
Other	88.67	86.81	84.96	*
Never vigorous or moderate	10.50	13.18	15.04	
**Alcohol consumption, n (%)**				
Not at all in the last 3 months	66.22	78.88	80.02	*
Less than once a month	12.56	9.54	6.28	*
Once or twice a month	5.97	4.73	4.94	*
More than once or twice a month	4.42	3.5	4.74	*
**Smoking status, n (%)**				
Smoker	14.21	12.77	*	*
Non smoker	50.77	82.18	*	*
**Affective/emotional disorder**^b^, **n (%)**	–	6.90	9.37	9.47
**At risk of severe deprivation**^a^, ** n (%)**	–	12.56	–	–

^a^It was only measured at wave 5 of the SHARE.

^b^It was not measured at wave 4 of the SHARE.

*It is not reported here due to more than 50% missing values in this variable. Note: the missing values were not due to nonresponse but simply because some of the participants received a condensed set of questions at wave 7.

**Pain intensity was not assessed in wave 4 of the SHARE. However, 75.56% participants reported being bothered by pain in back, knees, hips or other joint at wave 4.

### Direct effects

**Figure 1:**
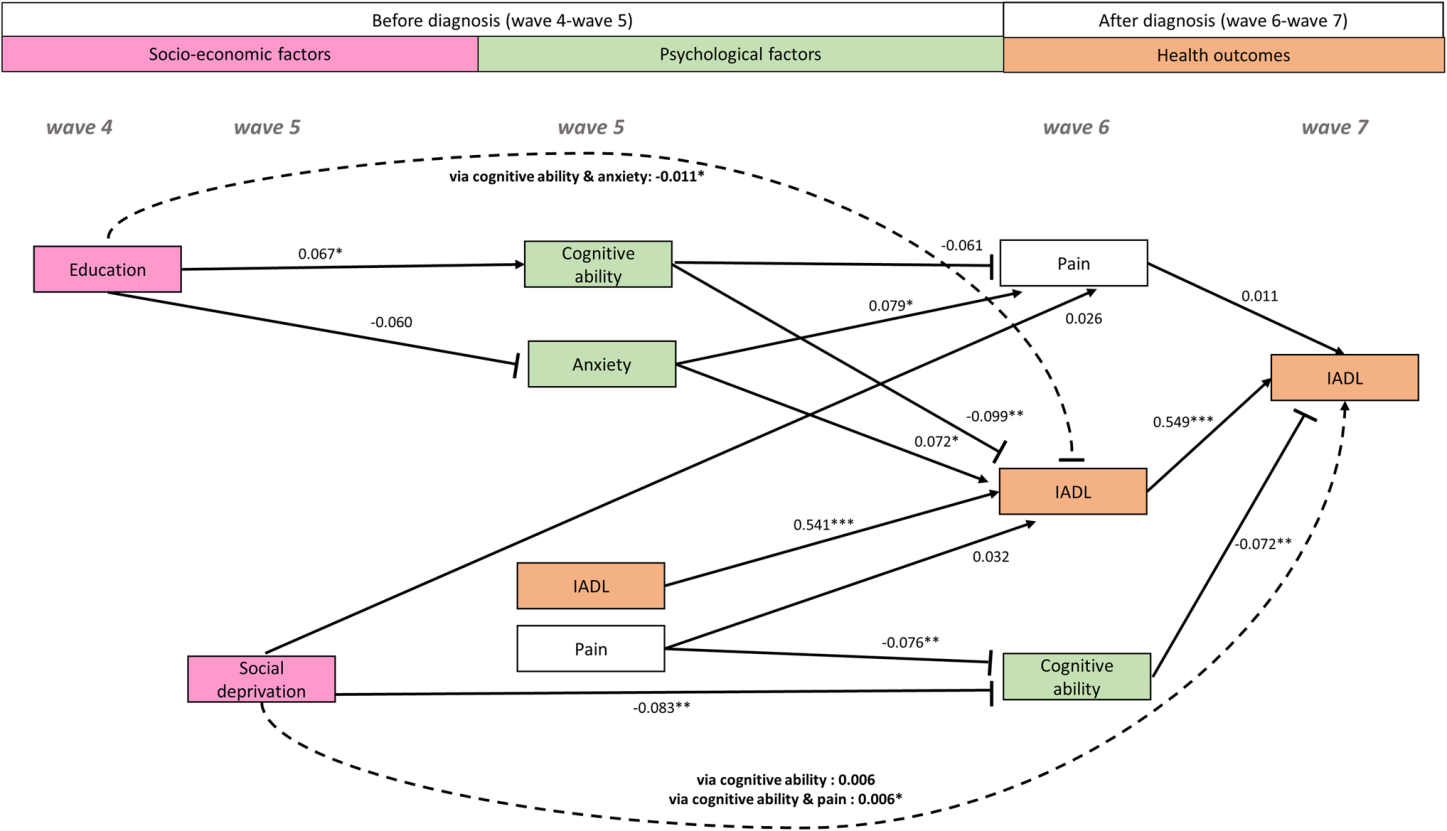
Illustration of key direct and indirect paths from socio-economic (education and social deprivation) and psychological factors (anxiety and cognitive ability) at waves 4 and 5 (before diagnosis) to health outcomes (pain and instrumental activities of daily living, IADL—note a higher IADL score indicates more difficulties with these activities) at wave 5, health outcomes and cognitive ability at wave 6 (following diagnosis) and IADL at wave 7 (after diagnosis). Inhibition arrows depict negative associations, whereas point arrows represent positive associations. Solid lines and dashed lines depict direct and indirect associations, respectively. Standardised effects and FDR-adjusted p-values are presented. Note: In path analysis, a variable can be both a predictor with respect to a variable and an outcome with regards to another variable as well as a mediator when testing for indirect effects^96^. For example, wave 6 cognitive ability is a predictor with regards to wave 7 IADL, an outcome with respect to wave 5 social deprivation and a mediator of the path from wave 5 social deprivation to wave 7 IADL. *p < 0.05, **p < 0.01, ***p < 0.001. n = 971.

The key findings are highlighted in the path model in Fig. 1 and in Supplementary Fig. S1 (for all outcomes of our analysis, see Supplementary Table S2). Hypothesis 1 stated that higher levels of pain will be related to reduced cognitive ability and higher anxiety after controlling for pain before diagnosis and confounders. Results from the path model show a direct association of higher anxiety before diagnosis with pain after diagnosis, such that greater anxiety is associated with more severe reports of pain. We find no significant association between cognitive ability before diagnosis and pain following diagnosis. Hypothesis 2 stated that better cognitive ability will be related to reduced pain levels and lower social deprivation after controlling for cognitive ability before diagnosis and confounders. In support of hypothesis 2, we find a direct association of higher levels of pain before diagnosis with reduced cognitive ability following diagnosis. In line with hypothesis 2, we find a direct association between social deprivation before diagnosis and cognitive ability following diagnosis, such that lower social deprivation is linked to better cognitive ability. Hypothesis 3 stated that greater limitations in activities of daily living will be associated with higher anxiety and poorer cognitive ability after controlling for limitations in activities of daily living before diagnosis and confounders. In support of hypothesis 3, results show that increased anxiety, before diagnosis, predicted greater limitations in activities of daily living (i.e., a higher IADL score) after diagnosis, whereas greater cognitive ability, before diagnosis, predicted reduced limitations in activities of daily living after diagnosis.

### Indirect effects

Hypothesis 4 stated that pain, cognitive ability and anxiety will act as the mechanisms indirectly linking socio-economic factors (social deprivation and educational attainment) to limitations in activities of daily living. Examining the indirect effects (Table 2), higher social deprivation, before diagnosis, predicted greater limitations in activities of daily living following diagnosis, through its negative effect on cognitive ability (wave 5 social deprivation → wave 6 cognitive ability → wave 7 IADL), suggesting that one mechanism underlying the link between social deprivation and limitations in activities of daily living is via cognitive ability. Looking at the total indirect effects, higher social deprivation before diagnosis was associated with greater limitations in activities of daily living after diagnosis (wave 7) via its association with reduced cognitive ability and higher pain levels at wave 6. In addition, higher educational attainment, prior to diagnosis was associated with lower limitations in activities of daily living after diagnosis via greater cognitive ability and lower anxiety before diagnosis (wave 5). Therefore, our results support hypothesis 4.

**Table 2:** Indirect paths predicting pain at wave 6 and IADL at waves 6 and 7 and their total indirect effects.

	SD coefficient	SE	CI lower	CI upper	P	FDR-adj. P
**Indirect paths**						
W5 Anxiety → W6 Cognitive ability → W7 IADL	0.000	0.002	− 0.003	0.004	0.778	0.821
W5 Anxiety → W6 Pain → W7 IADL	0.000	0.001	− 0.001	0.002	0.622	0.717
W4 Education → W5 Cognitive ability → W6 Cognitive ability	0.043	0.019	0.007	0.079	0.020*	0.043*
W4 Education → W5 Cognitive ability → W6 Pain	− 0.004	0.003	− 0.010	0.001	0.143	0.219
W4 Education→ W5 Anxiety → W6 Pain	− 0.005	0.003	− 0.011	0.001	0.134	0.207
W4 Education → W5 Cognitive ability → W6 IADL	− 0.007	0.003	− 0.013	0.000	0.049*	0.090
W4 Education → W5 Social Deprivation → W6 IADL	− 0.001	0.001	− 0.003	0.002	0.631	0.717
W4 Education → W5 Pain → W6 IADL	− 0.001	0.001	− 0.004	0.001	0.415	0.547
W4 Education → W5 Anxiety → W6 IADL	− 0.004	0.003	− 0.010	0.001	0.114	0.184
W5 Social deprivation → W6 Cognitive ability → W7 IADL	0.006	0.003	0.001	0.011	0.024*	0.050
W5 Social deprivation → W6 Pain → W7 IADL	0.000	0.001	− 0.001	0.002	0.677	0.756
**Total indirect effects**						
W5 Anxiety → W6 Cognitive ability + W6 Pain → W7 IADL	0.001	0.002	− 0.003	0.004	0.630	0.717
W4 Education→ W5 Cognitive ability + W5 Anxiety → W6 Pain	− 0.009	0.004	− 0.017	− 0.001	0.032*	0.065
W4 Education → W5 Pain + W5 Social deprivation → W6 IADL	− 0.002	0.002	− 0.005	0.002	0.346	0.479
W4 Education → W5 Cognitive ability + W5 Social deprivation → W6 IADL	− 0.007	0.004	− 0.014	0.000	0.040*	0.075
W4 Education → W5 Anxiety + W5 Social deprivation → W6 IADL	− 0.005	0.003	− 0.011	0.001	0.120	0.186
W4 Education→ W5 Cognitive ability + W5 Pain → W6 IADL	− 0.008	0.004	− 0.015	− 0.001	0.031*	0.065
W4 Education→ W5 Cognitive ability + W5 Anxiety → W6 IADL	− 0.011	0.004	− 0.019	− 0.003	0.011*	0.025*
W4 Education→ W5 Pain + W5 Anxiety → W6 IADL	− 0.005	0.003	− 0.011	0.000	0.071	0.125
W5 Social deprivation → W6 Cognitive ability + W6 Pain → W7 IADL	0.006	0.003	0.001	0.012	0.021*	0.044*

*W* wave, *SD* standardised, *SE* standard error, *CI* confidence interval, *FDR-adj.* false detection rate-adjusted, *IADL* independent activities of daily living.

*p < 0.05, **p < 0.01, ***p < 0.001. n = 971.

## Discussion

In a group of newly diagnosed osteoarthritis patients, we show that social deprivation before diagnosis predicted poorer cognitive ability after diagnosis. We also identified a link between social deprivation, prior to diagnosis and greater limitations in activities of daily living (as reflected by a higher IADL score) following diagnosis through its effect on reduced cognitive ability assessed early after diagnosis. This suggests that cognitive ability is a potential mechanism underlying the relationship between social deprivation and limitations in activities of daily living. We also demonstrated that higher levels of education before diagnosis might be protective against limitations in activities of daily living following diagnosis via better cognitive ability and lower anxiety.

To our knowledge, no previous study has assessed the interplay of social, cognitive and affective factors before a diagnosis of osteoarthritis and its impact on limitations in activities of daily living and pain severity following diagnosis. Previous longitudinal studies on the relationship between osteoarthritis pain, activities of daily living, deprivation and anxiety mostly included participants with longstanding osteoarthritis, making it hard to generalise their findings to those presenting with early disease^21,44–47^. For example, Hawker et al. (2011) constructed a path model to test the interrelationships between pain, depression, fatigue and disability over time, in participants with already established osteoarthritis^46^ and Harris et al*.* (2013) explored the impact of psychosocial risk factors (mainly perceived stress) on onset of osteoarthritis^48^. We extend these findings by exploring the mechanisms linking social deprivation and educational attainment with limitations in activities of daily living over time in newly diagnosed osteoarthritis patients taking into account the role of cognitive ability and anxiety in this relationship. While many aspects of social context can be difficult or impossible to change, understanding the pathways linking such aspects to health outcomes at the early disease stages also allows us to identify areas for health interventions.

The link between higher social deprivation and greater limitations in activities of daily living and the role of lower cognitive ability as a mediating mechanism may be explained by cognitive reserve theory. This theory suggests that continued stimulation of cognitive abilities, due to social engagement inhibits atrophy in these abilities and promotes better executive functioning in old age and is supported by previous research in humans^28–31^. At the same time, both higher pain levels and impaired cognitive ability might further reduce social participation and increase loneliness in seniors with and without arthritis^49,50^, creating a vicious cycle. It has been shown that impaired cognitive ability is associated with higher pain levels in arthritis^20,51^. Here, we found that pain and cognitive ability may act together early after diagnosis as a mechanism indirectly linking higher social deprivation with greater limitations in activities of daily living. In line with this finding, a previous cross-sectional study found that non-cancer pain and cognitive impairment are independently associated with limitations in IADL and limitations in IADL are even greater when both pain and cognitive impairment are present^52^. The mechanisms by which pain and impaired cognitive ability interact to exacerbate limitations in activities of daily living are unknown. A number of studies have demonstrated changes in brain morphology and connectivity, such as cerebral atrophy due to grey matter loss and bilateral hippocampal volume loss, in patients with chronic pain including with osteoarthritis^53,54^. It is possible, therefore, that the synergistic effect on limitations in activities of daily living of pain and impaired cognitive ability occurs via alterations in brain morphology.

It has been previously shown that low educational attainment is associated with higher pain levels and limitations in activities of daily living in osteoarthritis^23,24,32–36,55^. There is also a link between increased risk of osteoarthritis and less years of education seen in very large genetic studies^56,57^. Furthermore, education is an important determinant of cognitive ability in old age^58^. However, the pathways linking these factors in osteoarthritis are unknown. We found that education might be protective against limitations in activities of daily living via improved cognitive ability and lower anxiety. Bidirectional links between cognitive ability and affect have been demonstrated previously in arthritis^20,51^. However, we showed that these associations were influenced by previous educational attainment and that their interactions before diagnosis predicted activities vital to independence and self-care after diagnosis. Therefore, managing anxiety and potential problems with cognitive ability early in the disease course has potential clinical benefit in terms of activities of daily living. Cognitive-based interventions, involving the education about pain processing and false beliefs about movement can result in substantial improvements in disability and performance^59–63^, and could be considered as part of the arsenal of treatment options.

It is important to acknowledge the limitations of this study. The observed limitations in activities of daily living might be due to comorbid conditions, rather than osteoarthritis. However, we controlled for number of chronic conditions in our analyses and adjusted for previous IADL at each wave. Although this study’s model controlled to an extent for reverse causality between pain, IADL and cognitive ability, the model did not include social deprivation and anxiety in waves 6 and 7. Therefore, despite having assessed social deprivation before IADL and before diagnosis, the causality could be reversed and limitations in activities could affect social deprivation. Same-source bias can arise from use of perception-based measures (social deprivation index). However, perceived measures usually serve as good proxies of actual measures, as they are often highly correlated^64^. As we did not have information about osteoarthritis severity, despite controlling on disease duration, respondents may have had different levels of osteoarthritis pathology. We did not differentiate between the joints affected by osteoarthritis, and therefore we cannot exclude the possibility that our results may vary by joint. There is a scale by precision trade-off, which all large general cohorts have—we have a large longitudinal sample that is cross-cultural and representative and is used to address many questions, so the index of pain will, by definition, be general. It allows us to test general principles of cognitive ability, anxiety, pain, and deprivation that can be used to generate hypotheses for specific cohorts. There is also a possibility that some of the participants at the prediagnostic stage experience pain, which disrupts their ability to engage in day-to-day physical and cognitive tasks. Nevertheless, diagnosis is a critical time point, as previous studies have suggested that people often seek medical help when pain is severe enough and starts to interfere with meaningful day-to-day tasks^65–69^. In order to test the potential overlap between the social deprivation index and IADL, we first examined correlations between the items in both these instruments and then conducted additional analysis using an index of material deprivation assessed in wave 5 of the SHARE study and compared it with our original results using the social deprivation index. We found no identical items between the two scales and low correlations between the items (Supplementary Table S5) and we did not observe any major differences in the results between the two indices (Supplementary Tables S6 and S7), suggesting that our results are not due to conceptual overlap. Although we used self-reported osteoarthritis diagnosis, March and colleagues^70^ have found that self-reported physician diagnosed general arthritis has good congruency with clinically derived diagnoses. For the present study, two main pieces of evidence support that the measure of diagnosis is reliable. First, participants reported a diagnosis consistently at both wave 6 and wave 7. Second, we found that pain medication use increased significantly from wave 5 (before diagnosis) to wave 6 and wave 7 (after diagnosis), but did not differ significantly between the post-diagnostic waves, 6 and 7 (Supplementary Tables S8 and S9).

We show that higher social deprivation before osteoarthritis diagnosis is related to greater limitations in activities of daily living, after diagnosis with this effect partly mediated by impaired cognitive ability. We also show that, higher educational attainment before diagnosis may be protective against greater limitations in activity of daily living after diagnosis via better cognitive ability and lower anxiety before diagnosis. Therefore, improving cognitive ability and managing anxiety may mitigate the associations of social deprivation and low educational attainment with limitations in activities of daily living and may help to promote independence in patients with osteoarthritis.

## Methods

### Study population

**Figure 2:**
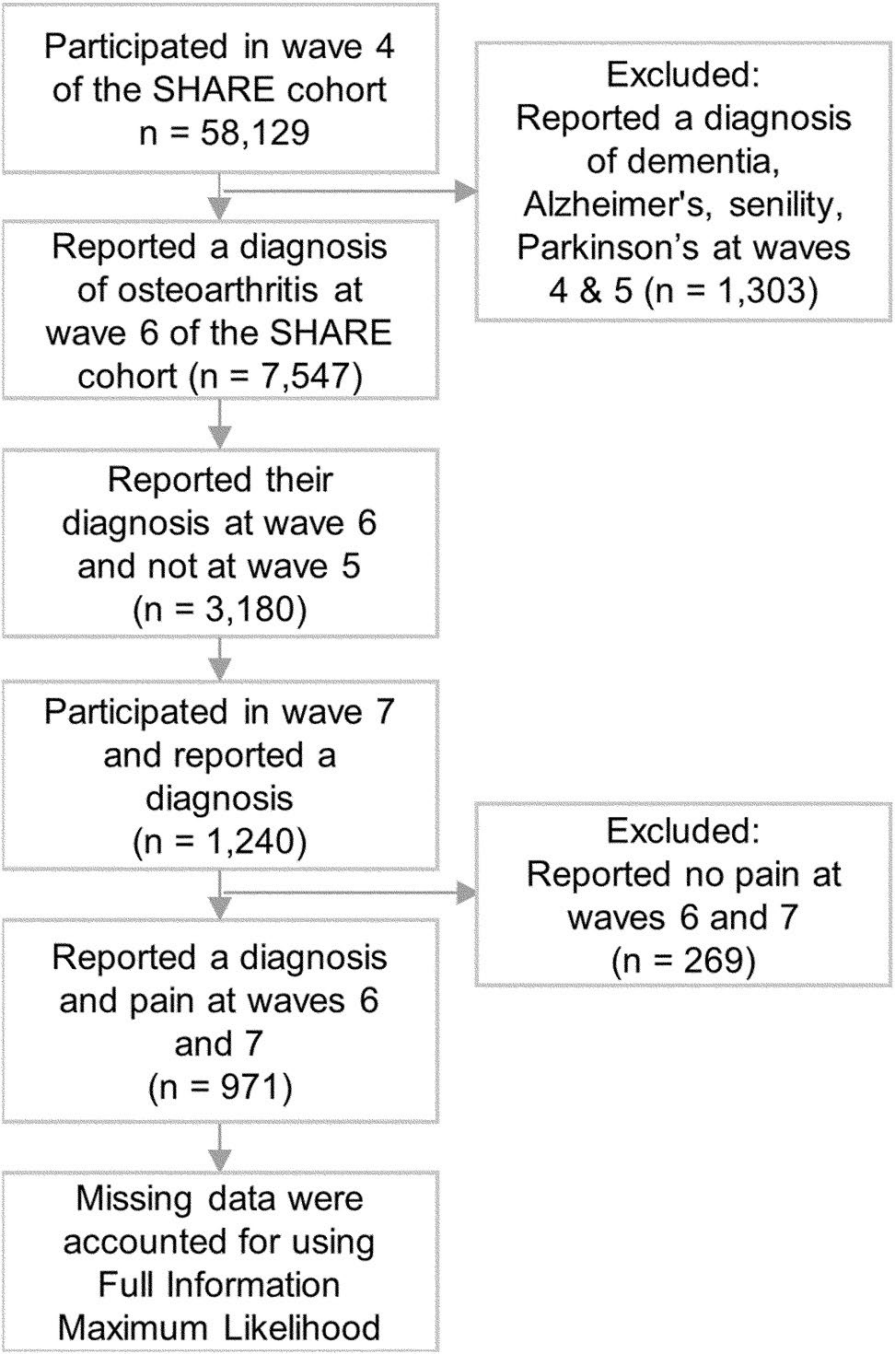
Flow chart of the assignment of respondents to the subsample analysed in this study.

Data were taken from waves 4 (2011)^71,72^, 5 (2013)^73,74^, 6 (2015)^75,76^, and 7 (2017)^75,77^ of the SHARE study, a multidisciplinary, cross-national, and longitudinal research project focusing on community-dwelling adults aged 50 or older^43^. The time between the waves was 2 years. We did not use data from waves 1 to 3, because they did not include specific information on osteoarthritis diagnosis (only on arthritis in general), and some of the key mechanistic measures, such as social deprivation and anxiety, were only assessed at wave 5. Detailed information about the entire SHARE project is available at www.share-project.org. SHARE respondents were included in our subsample if: (a) they had participated in wave 4 and did not report a diagnosis of dementia, Alzheimer’s disease, senility or Parkinson’s disease at waves 4 and 5 (i.e., before the diagnosis of osteoarthritis, see next inclusion criterion); (b) they reported a diagnosis of osteoarthritis at wave 6; (d) they did not report a diagnosis of osteoarthritis at wave 5; (e) they participated in wave 7; (f) continued to report a diagnosis of osteoarthritis at wave 7; and (g) they reported pain at both waves 6 and 7 (i.e., after the diagnosis of osteoarthritis) (Fig. 2). Of the 971 participants included in our analysis, none had missing data on measurements of pain and cognitive ability. Osteoarthritis diagnosis was assessed at all waves with the following question: “Has a doctor ever told you that you had/Do you currently have: Osteoarthritis? (With this we mean that a doctor has told you that you have this condition, and that you are either currently being treated for or bothered by this condition.)”^74^. The participants could have been diagnosed at any time in the period of 2 years after the wave 5 (2013) interview and before the wave 6 (2015) interview. Following diagnosis (wave 6), 479 (49.33%), 243 (25.03%), 424 (43.67%), 400 (41.19%), 56 (5.77%) participants reported to have back pain, hip pain, knee pain, pain in other joints, pain all over, respectively and 178 (18.33%), 524 (53.96%) and 269 (27.70%) reported mild, moderate and severe pain intensity levels, respectively.

### Ethics approval and consent to participate

The SHARE data collection procedures are subject to continuous ethics review by responsible ethics committees (University of Mannheim and Max Planck Society, Germany) as well as national ethics committees in participating countries. The reviews refer to all aspects of the project, from study design to informed consent. The reviews confirm the project agrees with international ethical standards, such as the Respect Code of Practice for Socio-Economic Research and the 'Declaration of Helsinki'.

For the purpose of the present study, appropriate permission was obtained for use of the SHARE data after successful application. The applicant is required to agree that use of the SHARE data will be purely for scientific purposes, provide appropriate information regarding their scientific alignment and agree to the SHARE Conditions of Use [http://www.share-project.org/data-access/share-conditions-of-use.html?L =]. Following successful application, the data can be accessed for free upon registration. The secondary analysis performed here required no additional ethical approvals.

### Variables

#### Cognitive ability

Cognitive ability was assessed at all waves and was based on multiple items: (1) immediate recall (participants were presented a list of 10 words and asked to repeat the words immediately; range = 0–10), (2) delayed recall (participants were asked for the list of 10 words after a delay; range = 0–10), (3) subtraction (participants were asked to mentally solve a subtraction task; range = 0–5), and (4) verbal fluency (participants were asked to produce as many animal names as possible within a given period of time; range = 0–100). We created a joint scale based on all items with a total score range of 0–125 (Cronbach’s alpha = 0.80, for wave 5 and Cronbach’s alpha = 0.76, for wave 6). The higher the score, the higher the participant’s cognitive ability^78^. These cognitive measures are validated and are widely used in many large longitudinal surveys^78,79^. Our calculated Cronbach’s alphas demonstrated good internal consistency reliability of the cognitive ability scale. We decided not to use a standardised score of cognitive ability due to the risks associated with their use in the analysis of longitudinal data^80,81^.

#### Pain

We constructed a pain score from two questions asked at all waves of the survey. Participants were asked whether they had been troubled by pain (yes/no). Those who replied positively were then asked to rate how bad their pain was most of the time (either mild, moderate or severe). The two variables were added to create a single score ranging from 1 (not troubled by pain) to 4 (troubled by severe pain), representing whether respondents were troubled by pain and how severe it was. This verbal rating scale used in the SHARE and other large longitudinal studies has been used widely in the pain literature^82^. Comparative and clinical trial studies^83–86^ have assessed the validity and reliability of this pain measure. Measurements using this verbal rating scale (0–4) are highly correlated with measurements using visual analogue or a numeric rating scale, and has similar precision for discriminating between treatments in osteoarthritis patients^85,86^.

#### Anxiety

In SHARE wave 5, five items were used to measure the severity of anxiety that was taken from the Beck Anxiety Inventory^87^. The respondents were asked about anxiety symptoms (“I had fear of the worst happening”, “I was nervous”, “I had a fear of dying”, “I felt my hands trembling”, and “I felt faint”) they experienced in the last 7 days and answer on a four point Likert scale (“never”, “hardly ever”, “some of the time”, and “most of the time”). We created a single anxiety scale by summing the scores of all five items to obtain an overall score, with higher scores indicating higher anxiety (Cronbach’s alpha = 0.69). These items have extensively been used in large longitudinal studies like the Health and Retirement Study and have been found to be valid for use in older populations^88^. Our calculated Cronbach’s alpha was 0.69.

#### Social deprivation index

A social deprivation index was provided in wave 5 of SHARE that was generated and validated by Michał et al*.* for the purpose of the SHARE study^89^. Briefly, this index was constructed using a battery of 15 questions related to participation in everyday life, social activities, and the quality of the neighbourhood following Chakravarty and D'Ambrosio^90^ and Levitas, et al.^25^. In order to combine different social deprivation items into a single index, Michał et al*.* computed the weight of each item based on a regression of the chosen items on the reported values of life satisfaction^89^. The most important elements of the index, those with the highest weight are: feeling left out of things, not feeling part of the neighbourhood, having no helpful people in the local area and waiting too long to see a doctor^89^.

#### Instrumental activities of daily living (IADL)

A modified version of IADL was used in SHARE^7,91^. IADL included seven activities in wave 5: “using a map to get around in a strange place”, “preparing a hot meal”, “shopping for groceries”, “making telephone calls”, “taking medications”, “doing work around the house or garden” and “managing money” with a total score ranging from 0 to 7. Two more items were added in waves 6 and 7: “leaving the house independently and accessing transportation services”, and “doing personal laundry”, resulting in nine items in total (score: 0–9). To account for the change in questions between waves 5, 6 and 7, we excluded from analysis the two extra items added at waves 6 and 7. A higher score indicates more difficulty with these activities (Cronbach’s alpha = 0.71, 0.80 and 0.86, for waves 5, 6 and 7, respectively).

#### Additional variables

Other variables included age (> 50 y.o.), gender, education measured with the International Standard Classification of Education (ISCED‐97)^92^, body mass index (BMI), smoking status (Currently smoking, Ex-smoker, Never smoked, and No response), alcohol consumption (How many drinks in 3 months), physical inactivity (Never moderate or vigorous activity and Other), number of chronic diseases (0–9), and marital status (Married and living together, Divorced, Widowed, and Other).

### Statistical analysis

Modelling was performed using R version 4.0.1. For path analysis the ‘lavaan’ package was used^93^. The missing mechanism of the SHARE data is assumed to be missing at random, and the level of dropout in this subsample is small (17.1%), thus, missing data were handled using full information maximum likelihood (FIML). We conducted sensitivity analyses using multiple imputations (m = 40). No significant differences between the two methods for handling missing data were found (see Supplementary Tables S11 and S12).

Path analysis was run using the pain and cognitive ability measurements at waves 5 and 6 and IADL at waves 5, 6 and 7. The path model was constructed such that the variance in IADL at wave x controlled for the variance in IADL at wave x-1, that is: the model looks at the variance in IADL once variance due to prior IADL is controlled. The same applies to pain and cognitive ability. Social deprivation and anxiety were added as predictor variables in the model at wave 5. The path model was adjusted for age, sex, education level, number of chronic diseases, BMI and alcohol consumption at baseline. Confounding variables were chosen due to their clinical relevance to chronic pain and cognitive ability based on findings from other studies^18^. Correlational analysis between the study variables was done to determine which variables were entered in the final model (see Supplementary Table S4 for correlational analyses). The standard errors were computed using the Delta method^94^. Our results were adjusted for multiple testing using false discovery rate (FDR < 0.05).

Model fit was assessed using the CFI, the TLI, the RMSEA and the SRMR. To determine acceptable fit we used the cut-off criteria proposed by Hu and Bentler^95^, who recommended an RMSEA lower than 0.06 and CFI and TLI greater than 0.95.

## Supplementary Information

Supplementary Information.

## Data Availability

This paper uses data from SHARE Waves 4, 5, 6 and 7 (DOIs: https://doi.org/10.6103/SHARE.w4.710, https://doi.org/10.6103/SHARE.w5.710, https://doi.org/10.6103/SHARE.w6.710, https://doi.org/10.6103/SHARE.w7.710), see Börsch-Supan et al.^43^ for methodological details.
