# Publisher Correction: The association of socio-economic and psychological factors with limitations in day-to-day activity over 7 years in newly diagnosed osteoarthritis patients

**DOI:** 10.1038/s41598-022-05907-3

**Published:** 2022-01-25

**Authors:** Afroditi Kouraki, Tobias Bast, Eamonn Ferguson, Ana M. Valdes

**Affiliations:** 1 School of Medicine, University of Nottingham, Nottingham, UK; 2 School of Psychology, University of Nottingham, University Park, Nottingham, UK; 3 Pain Centre Versus Arthritis, Academic Rheumatology, City Hospital, Nottingham, UK; 4 NIHR Nottingham Biomedical Research Centre, University of Nottingham, Nottingham, UK; 5 Neuroscience@Nottingham, University of Nottingham, Nottingham, UK

Correction to: *Scientific Reports* 10.1038/s41598-022-04781-3, published online 18 January 2022

The original version of this Article contained an error in the order of the Figures. Figures 1 and 2 were published as Figures 2 and 1. The Figure legends were correct.

The original Figures 1 and 2 and accompanying legends appear below.

**Figure 1:**
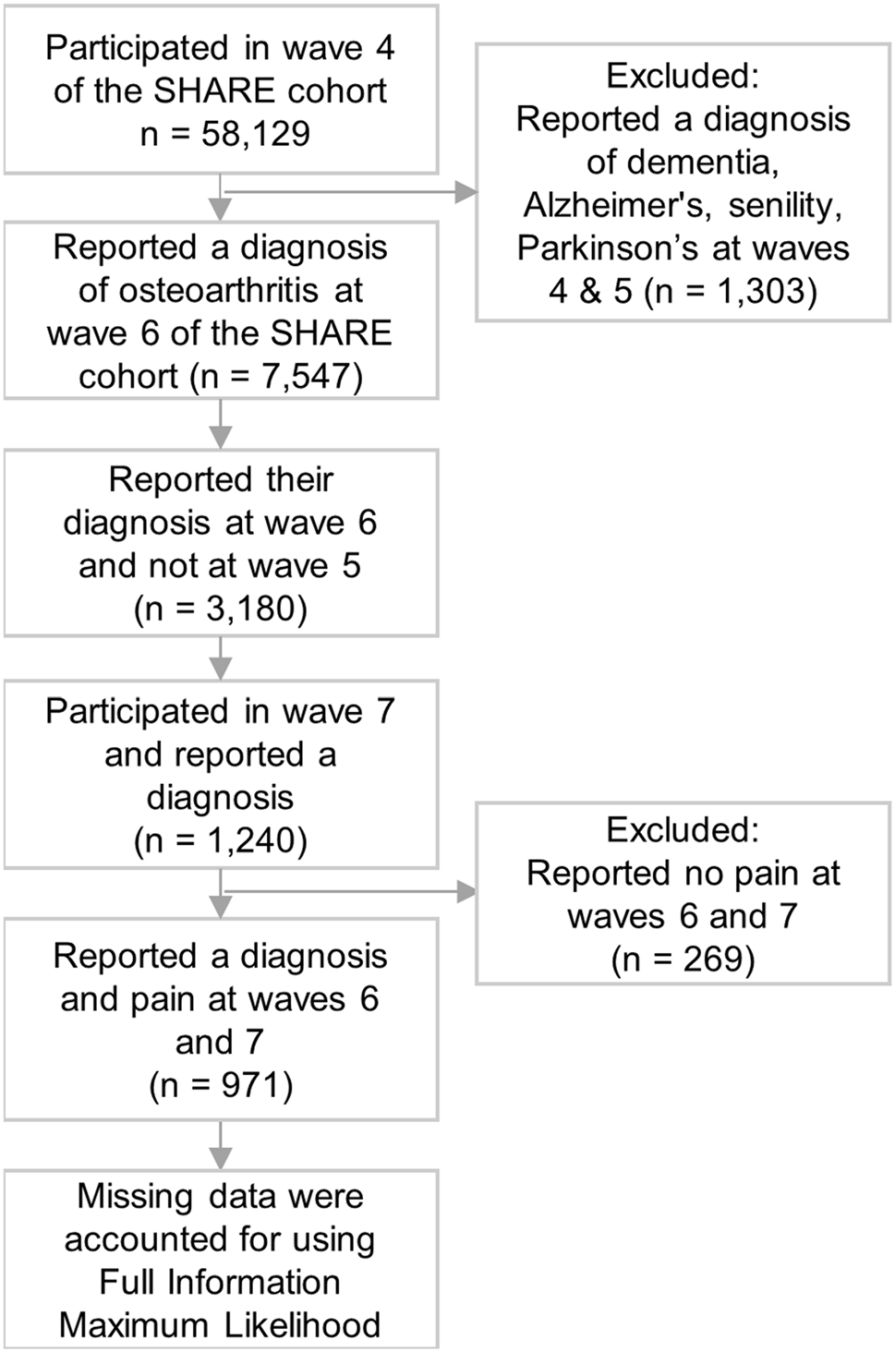
Illustration of key direct and indirect paths from socio-economic (education and social deprivation) and psychological factors (anxiety and cognitive ability) at waves 4 and 5 (before diagnosis) to health outcomes (pain and instrumental activities of daily living, IADL—note a higher IADL score indicates more difficulties with these activities) at wave 5, health outcomes and cognitive ability at wave 6 (following diagnosis) and IADL at wave 7 (after diagnosis). Inhibition arrows depict negative associations, whereas point arrows represent positive associations. Solid lines and dashed lines depict direct and indirect associations, respectively. Standardised effects and FDR-adjusted p-values are presented. Note: In path analysis, a variable can be both a predictor with respect to a variable and an outcome with regards to another variable as well as a mediator when testing for indirect effects^96^. For example, wave 6 cognitive ability is a predictor with regards to wave 7 IADL, an outcome with respect to wave 5 social deprivation and a mediator of the path from wave 5 social deprivation to wave 7 IADL. *p < 0.05, **p < 0.01, ***p < 0.001. n = 971.

**Figure 2:**
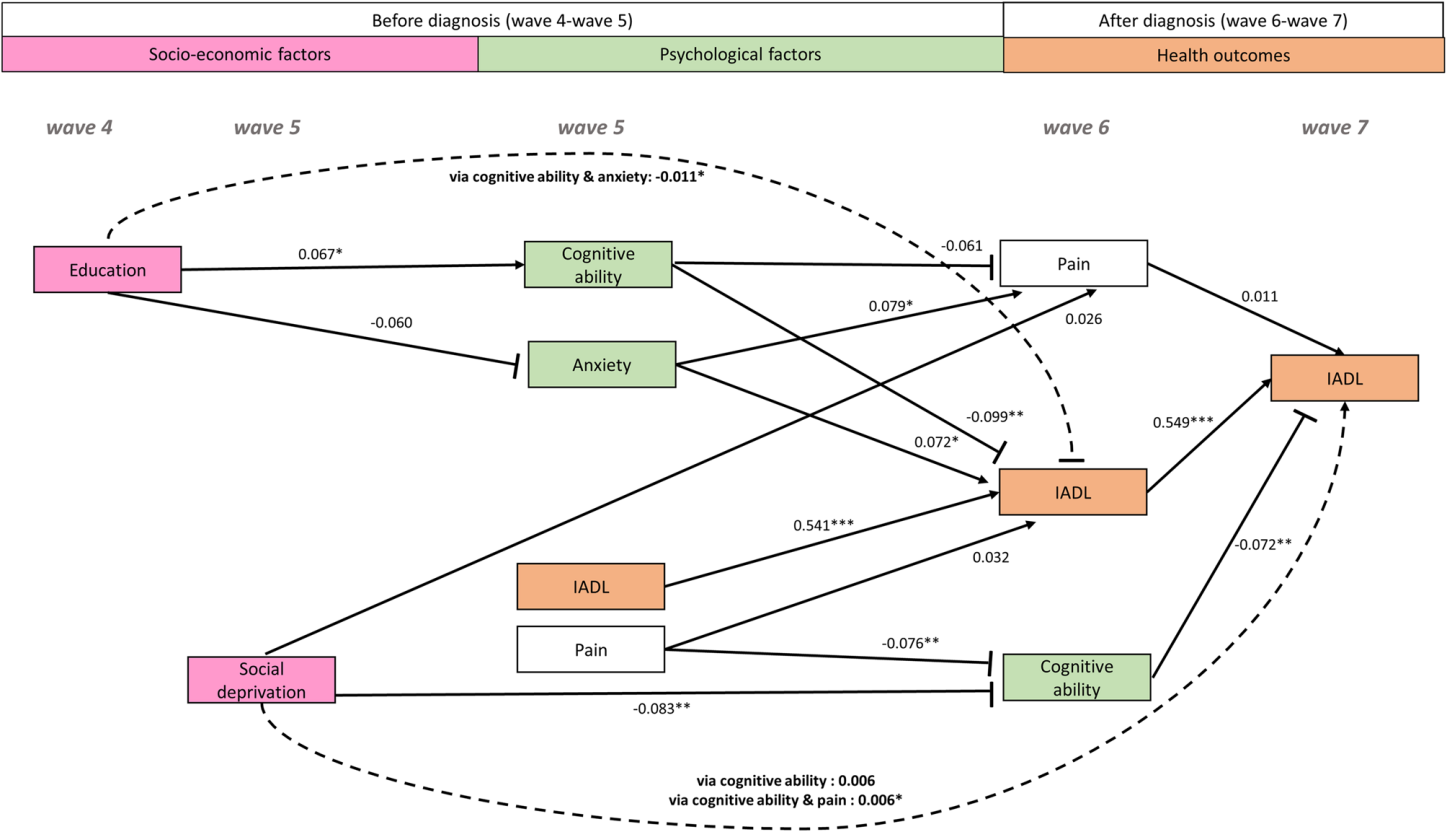
Flow chart of the assignment of respondents to the subsample analysed in this study.

The original article has been corrected.

