# Supplementary material for: The association of socio-economic and psychological factors with limitations in day-to-day activity over 7 years in newly diagnosed osteoarthritis patients
Source: Sci Rep. 2022 Jan 18;12:943. doi: 10.1038/s41598-022-04781-3 (PMC8766461; doi:10.1038/s41598-022-04781-3)
Supplement: Supplementary file 1 — Supplementary Information. [file 41598_2022_4781_MOESM1_ESM.pdf]

The association of socio-economic and psychological factors with limitations in day-to-day activity over 7 years in newly diagnosed osteoarthritis patients

Afroditi Kouraki[1, 4*], Tobias Bast[2, 3, 4, 5], Eamonn Ferguson[2, 3, 4], Ana M. Valdes[1, 3, 4]

1. School of Medicine, University of Nottingham, Nottingham, United Kingdom.

2. School of Psychology, University of Nottingham, University Park, Nottingham, United Kingdom.

3. Pain Centre Versus Arthritis, Academic Rheumatology, City Hospital, Nottingham, United Kingdom.

4. NIHR Nottingham Biomedical Research Centre, University of Nottingham, Nottingham, United Kingdom.

5. Neuroscience@Nottingham, University of Nottingham, Nottingham, United Kingdom

Table S1. *Model fit indices run with FIML*

Model fit indices	Statistic
**X^2 test of model fit**	82.323 (29) p <0.001
**RMSEA**	0.044
**CFI**	0.983
**TLI**	0.957
**X^2 test at baseline**	3218.297 (90) p <0.001
**SRMR**	0.026

Table S2. *Model standardised estimates of direct paths predicting pain and cognitive ability at wave 6 and IADL at waves 6 and 7*

Measure	Predictor	SD est.	S.E.	95% CI lower	95% CI upper	P	FDR-adj. P
**W5 Pain**	**W4 Gender**	-0.067	0.032	-0.129	-0.004	0.037*	0.071
**W5 Pain**	**W4 Age**	-0.019	0.033	-0.083	0.045	0.566	0.692
**W5 Pain**	**W4 Education**	-0.032	0.031	-0.094	0.029	0.300	0.414
**W5 Pain**	**W4 BMI**	0.043	0.033	-0.022	0.107	0.192	0.285
**W5 Pain**	**W4 Chronic diseases**	0.145	0.033	0.080	0.210	<0.001***	<0.001***
**W5 Pain**	**W4 Alcohol**	-0.008	0.040	-0.085	0.070	0.842	0.872
**W5 Cognitive ability**	**W4 Gender**	-0.027	0.030	-0.085	0.031	0.360	0.486
**W5 Cognitive ability**	**W4 Age**	-0.252	0.029	-0.309	-0.195	<0.001***	<0.001***
**W5 Cognitive ability**	**W4 Education**	0.067	0.029	0.011	0.123	0.019*	0.043*
**W5 Cognitive ability**	**W4 BMI**	-0.022	0.030	-0.082	0.037	0.459	0.572
**W5 Cognitive ability**	**W4 Chronic diseases**	-0.090	0.031	-0.151	-0.030	0.003**	0.008**
**W5 Cognitive ability**	**W4 Alcohol intake**	-0.079	0.039	-0.155	-0.003	0.042*	0.080
**W5 Anxiety**	**W4 Gender**	-0.054	0.033	-0.118	0.010	0.099	0.161
**W5 Anxiety**	**W4 Age**	0.005	0.033	-0.060	0.069	0.889	0.913
**W5 Anxiety**	**W4 Education**	-0.060	0.032	-0.122	0.002	0.058	0.106
**W5 Anxiety**	**W4 BMI**	0.014	0.033	-0.051	0.080	0.667	0.751
**W5 Anxiety**	**W4 Chronic diseases**	0.156	0.033	0.091	0.221	<0.001***	<0.001***
**W5 Anxiety**	**W4 Alcohol intake**	0.002	0.043	-0.083	0.086	0.969	0.977
**W5 Social deprivation**	**W4 Gender**	-0.059	0.033	-0.124	0.006	0.073	0.125
**W5 Social deprivation**	**W4 Age**	0.147	0.034	0.081	0.214	<0.001***	<0.001***
**W5 Social deprivation**	**W4 Education**	-0.017	0.032	-0.079	0.045	0.592	0.707
**W5 Social deprivation**	**W4 BMI**	0.003	0.034	-0.064	0.069	0.937	0.953
**W5 Social deprivation**	**W4 Chronic diseases**	0.099	0.035	0.031	0.167	0.004**	0.011*
**W5 Social deprivation**	**W4 Alcohol intake**	-0.011	0.042	-0.094	0.071	0.785	0.821

**W5 IADL**	**W4 Gender**	-0.053	0.030	-0.111	0.005	0.072	0.125
**W5 IADL**	**W4 Age**	0.220	0.029	0.162	0.277	<0.001***	<0.001***
**W5 IADL**	**W4 Education**	-0.009	0.029	-0.066	0.047	0.750	0.821
**W5 IADL**	**W4 BMI**	0.016	0.030	-0.043	0.075	0.591	0.707
**W5 IADL**	**W4 Chronic diseases**	0.166	0.031	0.107	0.226	<0.001***	<0.001***
**W5 IADL**	**W4 Alcohol intake**	-0.072	0.042	-0.154	0.009	0.081	0.135
**W6 Cognitive ability**	**W5 Cognitive ability**	0.643	0.020	0.604	0.682	<0.001***	<0.001***
**W6 Cognitive ability**	**W5 Pain**	-0.076	0.022	-0.118	-0.033	<0.001***	0.001**
**W6 Cognitive ability**	**W5 Anxiety**	-0.006	0.022	-0.050	0.037	0.777	0.821
**W6 Cognitive ability**	**W5 Social deprivation**	-0.083	0.024	-0.130	-0.036	0.001**	0.002**
**W6 Pain**	**W5 Pain**	0.248	0.030	0.188	0.307	<0.001***	<0.001***
**W6 Pain**	**W5 Cognitive ability**	-0.061	0.033	-0.125	0.003	0.060	0.107
**W6 Pain**	**W5 Anxiety**	0.079	0.032	0.016	0.141	0.014*	0.032*
**W6 Pain**	**W5 Social deprivation**	0.026	0.034	-0.041	0.092	0.449	0.566
**W6 IADL**	**W5 Pain**	0.032	0.024	-0.016	0.080	0.187	0.282
**W6 IADL**	**W5 Cognitive ability**	-0.099	0.027	-0.152	-0.047	<0.001***	0.001**
**W6 IADL**	**W5 IADL**	0.541	0.024	0.495	0.587	<0.001***	<0.001***
**W6 IADL**	**W5 Anxiety**	0.072	0.025	0.023	0.121	0.004**	0.010*
**W6 IADL**	**W5 Social deprivation**	0.033	0.030	-0.025	0.091	0.268	0.375
**W7 IADL**	**W6 Pain**	0.011	0.021	-0.031	0.052	0.618	0.717
**W7 IADL**	**W6 Cognitive ability**	-0.072	0.024	-0.119	-0.024	0.003**	0.008**
**W7 IADL**	**W6 IADL**	0.549	0.026	0.498	0.601	<0.001***	<0.001***
**W7 IADL**	**W5 Anxiety**	0.040	0.022	-0.004	0.083	0.074	0.125
**W7 IADL**	**W5 Social deprivation**	-0.001	0.024	-0.048	0.047	0.981	0.981

SD, standardised; SE, standard error; CI, confidence interval; FDR-adj., false detection rate-adjusted; W, wave; BMI, body mass index; IADL, instrumental activities of daily living. * = p < .05, ** = p < .01, *** = p < .001. n = 971

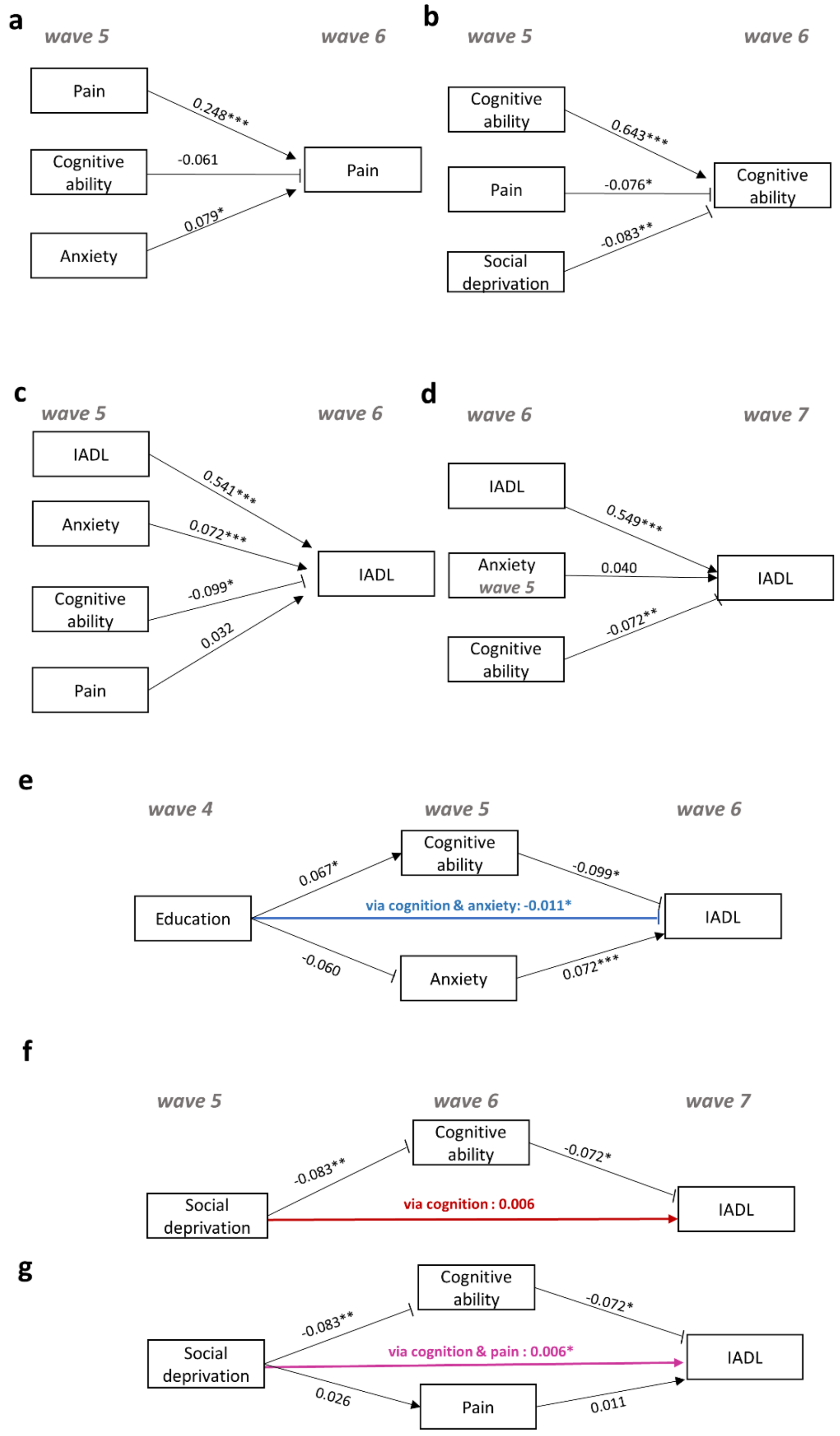

Figure S1. Illustration of direct and indirect paths based on our hypotheses*: (1) higher levels of pain will be related to impaired cognitive ability and higher anxiety after controlling for pain before diagnosis and confounders (panel a), (2) better cognitive ability will be related to reduced pain levels and lower social deprivation after controlling for cognitive ability before diagnosis and confounders (panel b), (3) more limitations in activities of daily living will be associated with higher anxiety and poorer cognitive ability after controlling for limitations in activities of daily living before diagnosis and confounders (panels c and d) and (4) pain, cognitive ability and anxiety will act as the mechanisms indirectly linking socio-economic factors (social deprivation and educational attainment) to limitations in activities of daily living (panels e, f, and g). IADL, instrumental activities of daily living. * = p < .05, ** = p < .01, *** = p < .001. n = 971*

Table S3. *R-Square values for predictor variables*

Variable	R-Square
**W5 Social deprivation**	0.043
**W5 Anxiety**	0.034
**W5 Cognitive ability**	0.220
**W6 Cognitive ability**	0.573
**W5 Pain**	0.071
**W6 Pain**	0.128
**W5 IADL**	0.226
**W6 IADL**	0.468
**W7 IADL**	0.590

W, wave; IADL, instrumental activities of daily living. n = 971

Table S4. *Bivariate, zero order correlations between the variables entered into the model.*

	**1**	**2**	**3**	**4**	**5**	**6**	**7**	**8**	**9**	**10**	**11**	**12**	**13**	**14**	**15**
**1. W5 Social deprivation**	1.000														
**2. W4 Education**	-0.019	1.000													
**3. W4 Age**	**0.150**	-0.035	1.000												
**4. W4 Sex**	0.055	-0.031	-0.012	1.000											
**5. W5Anxiety**	**0.302**	**-0.064**	0.043	**-0.068**	1.000										
**6. W5 Cognitive ability**	**-0.311**	**0.088**	**-0.332**	0.015	**-0.199**	1.000									
**7. W6 Cognitive ability**	**-0.316**	**0.104**	**-0.389**	**0.063**	**-0.177**	**0.735**	1.000								
**8. W5 Pain**	**0.150**	-0.046	0.036	**-0.089**	**0.168**	**-0.160**	**-0.197**	1.000							
**9. W6 Pain**	**0.117**	-0.060	**0.084**	**-0.070**	**0.163**	**-0.154**	**-0.160**	**0.300**	1.000						
**10. W6 IADL**	**0.261**	-0.057	**0.367**	**-0.097**	**0.214**	**-0.348**	**-0.383**	**0.179**	**0.194**	1.000					
**11. W7 IADL**	**0.237**	-0.044	**0.407**	-0.056	**0.212**	**-0.358**	**-0.392**	**0.148**	**0.181**	**0.743**	1.000				
**12. W5 IADL**	**0.297**	-0.038	**0.294**	**-0.078**	**0.188**	**-0.305**	**-0.311**	**0.201**	**0.206**	**0.648**	**0.595**	1.000			
**13. W4 BMI**	0.023	-0.016	-0.017	0.062	0.046	-0.042	-0.021	**0.083**	**0.130**	0.055	**0.073**	**0.070**	1		
**14. W4 Chronic diseases**	**0.121**	-0.017	**0.239**	**-0.081**	**0.165**	**-0.211**	**-0.155**	**0.188**	**0.215**	**0.271**	**0.307**	**0.284**	**0.217**	1.000	
**15. W4 Alcohol intake**	0.017	0.026	**0.127**	**-0.198**	0.017	**-0.108**	**-0.091**	0.002	0.022	0.011	0.025	-0.031	**-0.114**	**0.080**	1.000

W, wave; IADL, instrumental activities of daily living. Significant correlations are in bold. n = 971

Table S5. *Bivariate, zero order correlations between items of the social deprivation index and items of the IADL score.*

	Isolation	Course	Organisation	Trust	Doctor	Room	Feeling part	Vandalism	Clean area	Help in area	Bank access	Shop access	Pharmacy access	IT skills
**Using a map**	**-0.178**	**-0.084**	-0.051	-0.047	**-0.076**	**-0.183**	-0.015	0.007	0.041	-0.009	**0.183**	**0.173**	**0.173**	**0.235**
**Preparing hot meal**	**-0.108**	**-0.072**	**-0.061**	**-0.101**	**-0.068**	**-0.095**	-0.009	-0.079	-0.008	-0.019	**0.126**	**0.127**	**0.155**	**0.142**
**Grocery shopping**	**-0.211**	**-0.076**	-0.008	**-0.091**	**-0.084**	**-0.141**	-0.001	-0.031	0.048	-0.035	**0.217**	**0.220**	**0.213**	**0.184**
**Making calls**	**-0.186**	-0.036	**-0.058**	**-0.117**	**-0.076**	**-0.129**	-0.055	**-0.074**	-0.030	-0.032	0.054	**0.064**	**0.071**	**0.080**
**Taking medications**	**-0.131**	**-0.060**	**-0.059**	**-0.058**	**-0.102**	-0.036	-0.042	-0.066	-0.036	-0.013	-0.033	-0.049	-0.040	**0.079**
**Doing housework**	**-0.171**	**-0.083**	-0.048	-0.052	**-0.099**	**-0.074**	0.024	-0.028	-0.026	0.024	**0.175**	**0.184**	**0.151**	**0.164**
**Managing money**	**-0.181**	-0.051	-0.047	**-0.088**	**-0.134**	0.027	0.020	-0.034	0.027	0.007	**0.162**	**0.185**	**0.154**	**0.150**
**IADL total**	**-0.263**	**-0.110**	**-0.068**	**-0.111**	**-0.140**	**-0.141**	-0.003	-0.052	0.012	-0.010	**0.233**	**0.239**	**0.225**	**0.252**

Isolation: Feeling left out of things (Often=1, Sometimes=2, Rarely=3, Never=4), Course: Attended any course in the past 12 months (Yes=1, No=5), Organisation: Taken part in any organization in the past 12 months (Yes=1, No=5), Trust: People cannot be trusted. (0-10), Doctor: Waiting too long to see a doctor. (Yes=1, No=5), Room: How many rooms per person in household?, Feeling part: Feeling part of the local area. (Strongly agree=1, Agree=2, Disagree=3, Strongly disagree=4), Vandalism: Vandalism in the local area. (Strongly agree=1, Agree=2, Disagree=3, Strongly disagree=4), Clean area: Local area clean. (Strongly agree=1, Agree=2, Disagree=3, Strongly disagree=4), Help in area: Helpful people in local area. (Strongly agree=1, Agree=2, Disagree=3, Strongly disagree=4), Bank: Easy access to the nearest bank. (Very easy=1, Easy=2, Difficult=3, Very difficult=4), Shop: Easy access to the nearest shop. (Very easy=1, Easy=2, Difficult=3, Very difficult=4), Pharmacy: Easy access to the nearest pharmacy. (Very easy=1, Easy=2, Difficult=3, Very difficult=4). IT skills: Computer skills or never used a computer. (Excellent=1, Very good=2, Good=3, Fair=4, Poor=5, Never used=6), IADL, instrumental activities of daily living. Note: Literacy from social deprivation index is not reported due to >90 missing values, Significant correlations are in bold, n = 971

Table S6. *Comparison of standardized coefficients and standard errors (S.E.) from analyses based on two indices of deprivation: social and material*

		Social deprivation			Material deprivation[1]		
**Measure**	**Predictor**	**SD est.**	**S.E.**	**P**	**SD est.**	**S.E.**	**P**
**W5 Pain**	**W4 Gender**	-0.067	0.032	0.037*	-0.066	0.032	0.037*
**W5 Pain**	**W4 Age**	-0.019	0.033	0.566	0.003	0.032	0.936
**W5 Pain**	**W4 Education**	-0.032	0.031	0.300	-0.029	0.031	0.344
**W5 Pain**	**W4 BMI**	0.043	0.033	0.192	0.031	0.033	0.353
**W5 Pain**	**W4 Chronic diseases**	0.145	0.033	<0.001***	0.135	0.033	<0.001***
**W5 Pain**	**W4 Alcohol**	-0.008	0.040	0.842	-0.020	0.040	0.611
**W5 Cognitive ability**	**W4 Gender**	-0.027	0.030	0.360	-0.026	0.029	0.378
**W5 Cognitive ability**	**W4 Age**	-0.252	0.029	<0.001***	-0.300	0.028	<0.001***
**W5 Cognitive ability**	**W4 Education**	0.067	0.029	0.019*	0.062	0.029	0.031*
**W5 Cognitive ability**	**W4 BMI**	-0.022	0.030	0.459	0.001	0.030	0.962
**W5 Cognitive ability**	**W4 Chronic diseases**	-0.090	0.031	0.003**	-0.075	0.031	0.015*
**W5 Cognitive ability**	**W4 Alcohol intake**	-0.079	0.039	0.042*	-0.058	0.039	0.136
**W5 Anxiety**	**W4 Gender**	-0.054	0.033	0.099	-0.053	0.033	0.105
**W5 Anxiety**	**W4 Age**	0.005	0.033	0.889	0.004	0.033	0.905
**W5 Anxiety**	**W4 Education**	-0.060	0.032	0.058	-0.060	0.032	0.058
**W5 Anxiety**	**W4 BMI**	0.014	0.033	0.667	0.014	0.033	0.675
**W5 Anxiety**	**W4 Chronic diseases**	0.156	0.033	<0.001***	0.156	0.033	<0.001***
**W5 Anxiety**	**W4 Alcohol intake**	0.002	0.043	0.969	0.007	0.043	0.875
**W5 Deprivation**	**W4 Gender**	-0.059	0.033	0.073	-0.049	0.033	0.140
**W5 Deprivation**	**W4 Age**	0.147	0.034	<0.001***	-0.046	0.034	0.172
**W5 Deprivation**	**W4 Education**	-0.017	0.032	0.592	-0.036	0.032	0.258
**W5 Deprivation**	**W4 BMI**	0.003	0.034	0.937	0.094	0.033	0.005**
**W5 Deprivation**	**W4 Chronic diseases**	0.099	0.035	0.004**	0.148	0.034	<0.001***
**W5 Deprivation**	**W4 Alcohol intake**	-0.011	0.042	0.785	0.076	0.046	0.099
**W5 IADL**	**W4 Gender**	-0.053	0.030	0.072	-0.057	0.030	0.057
**W5 IADL**	**W4 Age**	0.220	0.029	<0.001***	0.258	0.029	<0.001***
**W5 IADL**	**W4 Education**	-0.009	0.029	0.750	-0.006	0.029	0.831
**W5 IADL**	**W4 BMI**	0.016	0.030	0.591	0.004	0.031	0.905
**W5 IADL**	**W4 Chronic diseases**	0.166	0.031	<0.001***	0.163	0.031	<0.001***

**W5 IADL**	**W4 Alcohol intake**	-0.072	0.042	0.081	-0.081	0.043	0.058
**W6 Cognitive ability**	**W5 Cognitive ability**	0.643	0.020	<0.001***	0.640	0.020	<0.001***
**W6 Cognitive ability**	**W5 Pain**	-0.076	0.022	<0.001***	-0.072	0.022	0.001**
**W6 Cognitive ability**	**W5 Anxiety**	-0.006	0.022	0.777	-0.010	0.022	0.648
**W6 Cognitive ability**	**W5 Deprivation**	-0.083	0.024	0.001**	-0.083	0.024	<0.001***
**W6 Pain**	**W5 Pain**	0.248	0.030	<0.001***	0.248	0.030	<0.001***
**W6 Pain**	**W5 Cognitive ability**	-0.061	0.033	0.060	-0.066	0.032	0.040*
**W6 Pain**	**W5 Anxiety**	0.079	0.032	0.014*	0.083	0.032	0.009**
**W6 Pain**	**W5 Deprivation**	0.026	0.034	0.449	0.009	0.033	0.783
**W6 IADL**	**W5 Pain**	0.032	0.024	0.187	0.029	0.024	0.243
**W6 IADL**	**W5 Cognitive ability**	-0.099	0.027	<0.001***	-0.094	0.027	0.001**
**W6 IADL**	**W5 IADL**	0.541	0.024	<0.001***	0.543	0.023	<0.001***
**W6 IADL**	**W5 Anxiety**	0.072	0.025	0.004**	0.070	0.025	0.004**
**W6 IADL**	**W5 Deprivation**	0.033	0.030	0.268	0.048	0.028	0.087
**W7 IADL**	**W6 Pain**	0.011	0.021	0.618	0.010	0.021	0.650
**W7 IADL**	**W6 Cognitive ability**	-0.072	0.024	0.003**	-0.064	0.024	0.009**
**W7 IADL**	**W6 IADL**	0.549	0.026	<0.001***	0.547	0.026	<0.001***
**W7 IADL**	**W5 Anxiety**	0.040	0.022	0.074	0.035	0.022	0.116
**W7 IADL**	**W5 Deprivation**	-0.001	0.024	0.981	0.028	0.024	0.234

[1]An index of material deprivation was assessed in Wave 5 of the SHARE and includes 11 items that cover aspects of the economic circumstances of households, such as the ability to afford to eat meat or fruit more often than three times per week, the affordability of a number of specific items such as groceries and holidays away from home, the necessity to limit expenses on a number of items, such as shoes or heating to keep living costs down, and the inability to see a doctor because of cost.

SD, standardised; SE, standard error; W, wave; IADL, instrumental activities of daily living. * = p < .05, ** = p < .01, *** = p < .001. n=971

Table S7. *Comparison of standardized coefficients and standard errors (S.E.) from analyses based on two indices of deprivation: social and material*

	**Social deprivation**			**Material deprivation[1]**		
**Indirect paths**	**SD est.**	**S.E.**	**P**	**SD est.**	**S.E.**	**p**
W5 Anxiety → W6 Cognitive ability → W7IADL	0.000	0.002	0.778	0.001	0.001	0.653
W5 Anxiety → W6 Pain → W7 IADL	0.000	0.001	0.622	0.000	0.001	0.655
W4 Education → W5 Cognitive ability → W6 Cognitive ability	0.043	0.019	0.020*	0.039	0.018	0.032*
W4 Education → W5 Cognitive ability → W6 Pain	-0.004	0.003	0.143	-0.004	0.003	0.138
W4 Education→ W5 Anxiety → W6 Pain	-0.005	0.003	0.134	-0.005	0.003	0.125
W4 Education → W5 Cognitive ability → W6 IADL	-0.007	0.003	0.049*	-0.006	0.003	0.068
W4 Education → W5 Deprivation → W6 IADL	-0.001	0.001	0.631	-0.002	0.002	0.347
W4 Education → W5 Pain → W6 IADL	-0.001	0.001	0.415	-0.001	0.001	0.462
W4 Education → W5 Anxiety → W6 IADL	-0.004	0.003	0.114	-0.004	0.003	0.115
W5 Deprivation → W6 Cognitive ability → W7 IADL	0.006	0.003	0.024*	0.005	0.003	0.036*
W5 Deprivation → W6 Pain → W7 IADL	0.000	0.001	0.677	0.000	0.000	0.814
**Total indirect effects**	**SD est.**	**S.E.**	**P**	**SD est.**	**S.E.**	**p**
W5 Anxiety → W6 Cognitive ability+W6 Pain → W7 IADL	0.001	0.002	0.630	0.001	0.002	0.543
W4 Education→ W5 Cognitive ability +W5 Anxiety → W6 Pain	-0.009	0.004	0.033*	-0.009	0.004	0.029*
W4 Education → W5 Pain+W5 Deprivation → W6 IADL	-0.002	0.002	0.351	-0.003	0.002	0.226
W4 Education → W5 Cognitive ability+W5 Deprivation → W6IADL	-0.007	0.004	0.040*	-0.007	0.004	0.033*
W4 Education → W5 Anxiety+W5 Deprivation → W6 IADL	-0.005	0.003	0.118	-0.006	0.003	0.079
W4 Education→ W5 Cognitive ability+W5 Pain → W6 IADL	-0.008	0.004	0.032*	-0.007	0.003	0.048*
W4 Education→ W5 Cognitive ability+W5 Anxiety → W6 IADL	-0.011	0.004	0.011*	-0.010	0.004	0.015*
W4 Education→ W5 Pain+W5 Anxiety → W6 IADL	-0.005	0.003	0.072	-0.005	0.003	0.079
W5 Deprivation → W6 Cognitive ability+W6 Pain → W7 IADL	0.006	0.003	0.021*	0.005	0.003	0.034*

[1]An index of material deprivation was assessed in Wave 5 of the SHARE and includes 11 items that cover aspects of the economic circumstances of households, such as the ability to afford to eat meat or fruit more often than three times per week, the affordability of a number of specific items such as groceries and holidays away from home, the necessity to limit expenses on a number of items, such as shoes or heating to keep living costs down, and the inability to see a doctor because of cost.

SD, standardised; SE, standard error; W, wave; IADL, instrumental activities of daily living.* = p < .05, ** = p < .01, *** = p < .001. n=971

Table S8. *One-way ANOVA to compare pain medication use across wave 5, 6 and 7.*

	Degrees of freedom	Sum of squares	Mean Square	F value	P>F
**wave**	2	19.900	9.966	21.100	<0.001***
**Residuals**	3769	1780	0.472		

Data on pain medication use were collected at all waves by asking participants, "Do you currently take drugs at least once a week for joint pain or for joint inflammation?" The question does not distinguish between over-the counter or prescription drugs. * = p < .05, ** = p < .01, *** = p < .001. n=969 (2 observations were deleted due to missingness)

Table S9. *Post hoc multiple comparisons of mean pain medication use between the different waves with Tukey test*

	Mean difference	Lower Limit 95% Confidence Interval	Upper Limit 95% Confidence Interval	FDR-adj. P value
**W6 Pain Medication-W5 Pain Medication**	0.149	0.084	0.213	<0.001***
**W7 Pain Medication-W5 Pain Medication**	0.159	0.095	0.223	<0.001***
**W7 Pain Medication-W6 Pain Medication**	0.011	-0.054	0.075	0.700

Data on pain medication use were collected at all waves by asking participants, "Do you currently take drugs at least once a week for joint pain or for joint inflammation?" The question does not distinguish between over-the counter or prescription drugs. W, wave; FDR-adj., False discovery rate-adjusted, * = p < .05, ** = p < .01, *** = p < .001. n=971

Table S10. *Number of missing data per variable*

Variable	Number of missing data
**W4BMI**	33
**W4Chronic diseases**	1
**W4Alcohol intake**	315
**W5Social deprivation**	83
**W4Education**	11
**W4Age**	0
**W4Sex**	0
**W5Anxiety**	0
**W5Cognitive ability**	0
**W6Cognitve ability**	0
**W5Pain**	0
**W6Pain**	0
**W6IADL**	0
**W7IADL**	5
**W5IADL**	0

W, wave; BMI, body mass index; IADL, instrumental activities of daily living. n = 971

Table S11. *Comparison of unstandardized coefficients and standard errors from analyses based on two methods for handling missing data: full information maximum likelihood (FIML) and multiple imputation (MI)*

		**FIML (n = 971)**			**MI (m = 40)**		
**Measure**	**Predictor**	**Est.**	**S.E.**	**P**	**Est.**	**S.E.**	**P**
**W5 Pain**	**W4 Gender**	-0.165	0.079	0.037*	-0.168	0.081	0.039*
**W5 Pain**	**W4 Age**	-0.002	0.004	0.566	-0.002	0.004	0.616
**W5 Pain**	**W4 Education**	-0.005	0.005	0.300	-0.005	0.005	0.317
**W5 Pain**	**W4 BMI**	0.009	0.007	0.192	0.009	0.007	0.203
**W5 Pain**	**W4 Chronic diseases**	0.099	0.023	<0.001***	0.099	0.024	<0.001***
**W5 Pain**	**W4 Alcohol**	-0.009	0.047	0.842	-0.014	0.041	0.727
**W5 Cognitive ability**	**W4 Gender**	-0.723	0.791	0.360	-0.691	0.808	0.393
**W5 Cognitive ability**	**W4 Age**	-0.319	0.038	<0.001***	-0.323	0.039	<0.001***
**W5 Cognitive ability**	**W4 Education**	0.104	0.045	0.019*	0.104	0.046	0.024*
**W5 Cognitive ability**	**W4 BMI**	-0.050	0.068	0.459	-0.046	0.068	0.501
**W5 Cognitive ability**	**W4 Chronic diseases**	-0.668	0.228	0.003**	-0.693	0.234	0.003**
**W5 Cognitive ability**	**W4 Alcohol intake**	-1.023	0.505	0.042*	-1.030	0.405	0.011*
**W5 Anxiety**	**W4 Gender**	-0.361	0.219	0.099	-0.359	0.224	0.109
**W5 Anxiety**	**W4 Age**	0.001	0.010	0.889	0.001	0.011	0.902
**W5 Anxiety**	**W4 Education**	-0.023	0.012	0.058	-0.024	0.013	0.064
**W5 Anxiety**	**W4 BMI**	0.008	0.019	0.667	0.008	0.019	0.692
**W5 Anxiety**	**W4 Chronic diseases**	0.288	0.062	<0.001***	0.290	0.064	<0.001***
**W5 Anxiety**	**W4 Alcohol intake**	0.005	0.140	0.969	0.008	0.112	0.941
**W5 Social deprivation**	**W4 Gender**	-1.979	1.108	0.073	-1.934	1.102	0.079
**W5 Social deprivation**	**W4 Age**	0.232	0.054	<0.001***	0.222	0.053	<0.001***
**W5 Social deprivation**	**W4 Education**	-0.033	0.061	0.592	-0.028	0.063	0.651
**W5 Social deprivation**	**W4 BMI**	0.008	0.095	0.937	0.008	0.093	0.928
**W5 Social deprivation**	**W4 Chronic diseases**	0.907	0.321	0.004**	0.853	0.316	0.007**
**W5 Social deprivation**	**W4 Alcohol intake**	-0.185	0.680	0.785	-0.284	0.553	0.607
**W5 IADL**	**W4 Gender**	-0.115	0.064	0.072	-0.125	0.065	0.056
**W5 IADL**	**W4 Age**	0.022	0.003	<0.001***	0.023	0.003	<0.001***
**W5 IADL**	**W4 Education**	-0.001	0.004	0.750	-0.001	0.004	0.744
**W5 IADL**	**W4 BMI**	0.003	0.005	0.591	0.002	0.006	0.654

**W5 IADL**	**W4 Chronic diseases**	0.100	0.019	<0.001***	0.101	0.019	<0.001***
**W5 IADL**	**W4 Alcohol intake**	-0.076	0.044	0.081	-0.099	0.033	0.003**
**W6 Cognitive ability**	**W5 Cognitive ability**	0.622	0.023	<0.001***	0.624	0.023	<0.001***
**W6 Cognitive ability**	**W5 Pain**	-0.789	0.225	<0.001***	-0.796	0.231	0.001**
**W6 Cognitive ability**	**W5 Anxiety**	-0.024	0.086	0.777	-0.027	0.089	0.762
**W6 Cognitive ability**	**W5 Social deprivation**	-0.065	0.019	0.001**	-0.061	0.019	0.001**
**W6 Pain**	**W5 Pain**	0.152	0.019	<0.001***	0.152	0.020	<0.001***
**W6 Pain**	**W5 Cognitive ability**	-0.003	0.002	0.060	-0.004	0.002	0.064
**W6 Pain**	**W5 Anxiety**	0.018	0.007	0.014*	0.018	0.007	0.018*
**W6 Pain**	**W5 Social deprivation**	0.001	0.002	0.449	0.001	0.002	0.459
**W6 IADL**	**W5 Pain**	0.045	0.034	0.187	0.046	0.035	0.192
**W6 IADL**	**W5 Cognitive ability**	-0.013	0.004	<0.001***	-0.013	0.004	<0.001***
**W6 IADL**	**W5 IADL**	0.880	0.043	<0.001***	0.884	0.044	<0.001***
**W6 IADL**	**W5 Anxiety**	0.038	0.013	0.004**	0.039	0.014	0.004**
**W6 IADL**	**W5 Social deprivation**	0.003	0.003	0.268	0.002	0.003	0.423
**W7 IADL**	**W6 Pain**	0.030	0.060	0.618	0.030	0.061	0.629
**W7 IADL**	**W6 Cognitive ability**	-0.012	0.004	0.003**	-0.012	0.004	0.003**
**W7 IADL**	**W6 IADL**	0.669	0.035	<0.001***	0.669	0.035	<0.001***
**W7 IADL**	**W6 Anxiety**	0.025	0.014	0.074	0.026	0.015	0.079
**W7 IADL**	**W5 Social deprivation**	0.000	0.003	0.981	-0.001	0.003	0.864

Model fit indices with MI: RMSEA = 0.044, 95% CI 0.033- 0.055, SRMR = 0.031, CFI = 0.980, TLI = 0.950

Est., estimate; SE, standard error; W, wave; IADL, instrumental activities of daily living. n = 971

Table S12. *Comparison of standardized coefficients and standard errors from analyses based on two methods for handling missing data: FIML and MI*

**Indirect paths**	**FIML SD est.**	**FIML S.E.**	**P**	**MI SD est.**	**MI S.E.**	**P**
W5 Anxiety → W6 Cognitive ability → W7 IADL	0.000	0.001	0.778	0.000	0.001	0.762
W5 Anxiety → W6 Pain → W7 IADL	0.001	0.002	0.622	0.001	0.002	0.617
W4 Education → W5 Cognitive ability → W6 Cognitive ability	0.065	0.028	0.020*	0.065	0.029	0.024*
W4 Education → W5 Cognitive ability → W6 Pain	-0.000	0.000	0.144	0.000	0.000	0.163
W4 Education→ W5 Anxiety → W6 Pain	-0.000	0.000	0.134	0.000	0.000	0.150
W4 Education → W5 Cognitive ability → W6 IADL	-0.001	0.001	0.049*	-0.001	0.001	0.047*
W4 Education → W5 Social Deprivation → W6 IADL	-0.000	0.000	0.631	0.000	0.000	0.801
W4 Education → W5 Pain → W6 IADL	-0.000	0.000	0.415	0.000	0.000	0.419
W4 Education → W5 Anxiety → W6 IADL	-0.001	0.001	0.115	-0.001	0.001	0.109
W5 Social deprivation → W6 Cognitive ability → W7 IADL	0.001	0.000	0.024*	0.001	0.000	0.021*
W5 Social deprivation → W6 Pain → W7 IADL	0.000	0.000	0.677	0.000	0.000	0.597
**Total indirect effects**	**FIML SD est.**	**FIML S.E.**	**P**	**MI SD est.**	**MI S.E.**	**P**
W5 Anxiety → W6 Cognitive ability+W6 Pain → W7 IADL	0.001	0.002	0.570	0.001	0.002	0.557
W4 Education→ W5 Cognitive ability+W5 Anxiety → W6 Pain	-0.001	0.000	0.033*	-0.001	0.000	0.040*
W4 Education → W5 Pain+W5 Social deprivation → W6 IADL	-0.000	0.000	0.351	0.000	0.000	0.451
W4 Education → W5 Cognitive ability+W5 Social deprivation → W6 IADL	-0.001	0.001	0.040*	-0.001	0.001	0.048*
W4 Education → W5 Anxiety+W5 Social deprivation → W6 IADL	-0.001	0.001	0.119	-0.001	0.001	0.143
W4 Education→ W5 Cognitive ability+W5 Pain → W6 IADL	-0.002	0.001	0.032*	-0.002	0.001	0.032*
W4 Education→ W5 Cognitive ability+W5 Anxiety → W6 IADL	-0.002	0.001	0.011*	-0.002	0.001	0.009**
W4 Education→ W5 Pain+W5 Anxiety → W6 IADL	-0.001	0.001	0.072	-0.001	0.001	0.069
W5 Social deprivation → W6 Cognitive ability+W6 Pain → W7 IADL	0.001	0.000	0.020*	0.001	0.000	0.017*

Model fit indices with MI: RMSEA = 0.044, 95% CI 0.033- 0.055, SRMR = 0.031, CFI = 0.980, TLI = 0.950. Est., estimate; SE, standard error; W, wave; IADL, instrumental activities of daily living. n = 971

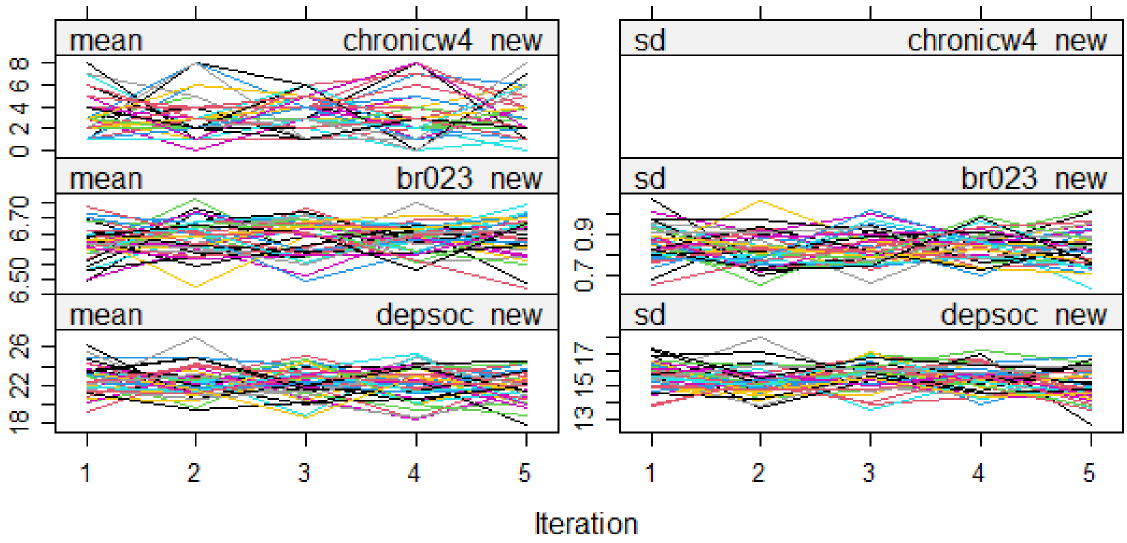

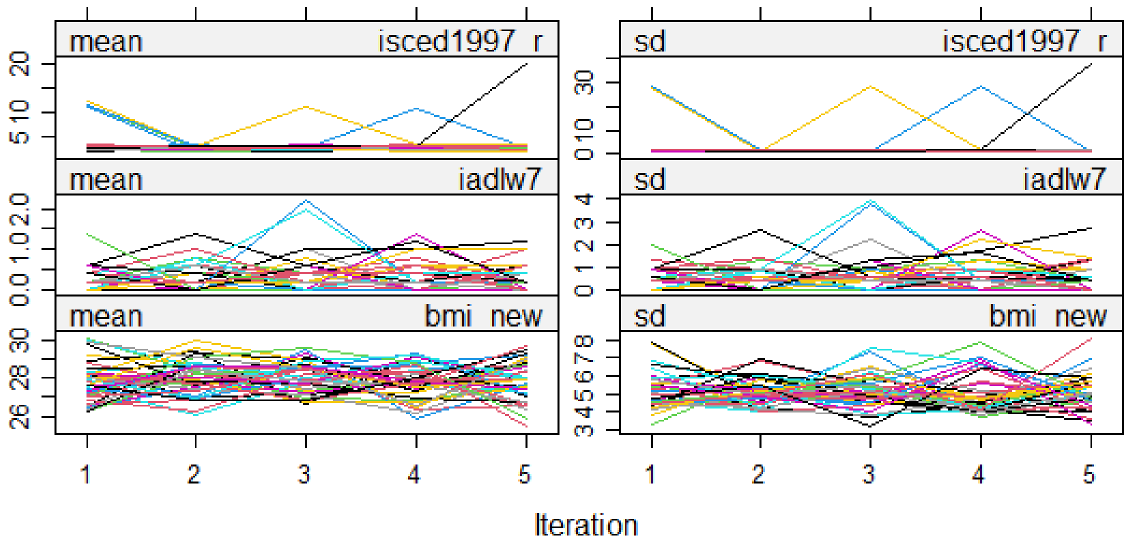

Figure S2. *Convergence plots. The plots show the mean (left) and standard deviation (right) of the imputed values for each of the imputed variables. w, wave; chronicw4_new, number of diagnosed chronic diseases; br023_new, alcohol intake; depsoc_new, social deprivation; isced1997_r, educational attainment; iadl, instrumental of daily living; bmi_new, body mass index. m=40*

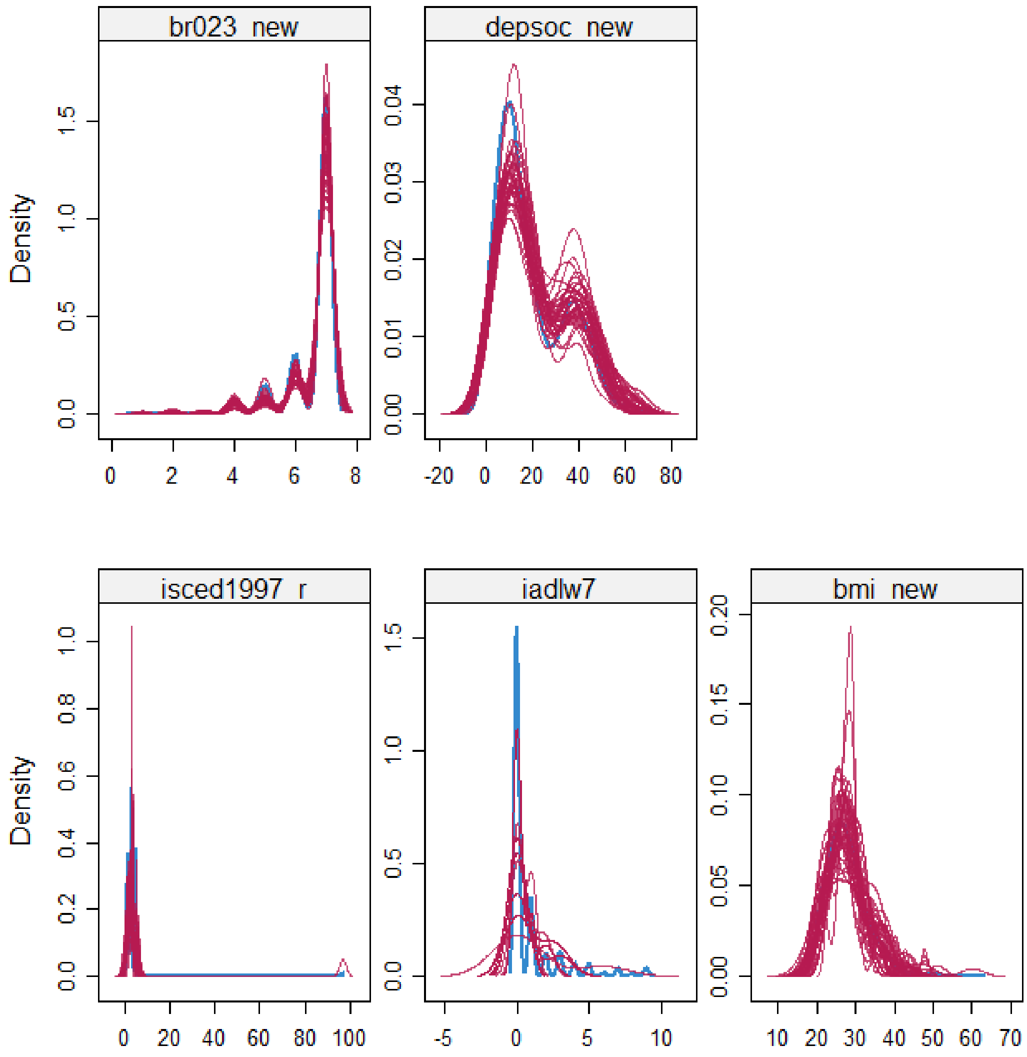

Figure S3. *Density plots. Red lines depict imputed data while the blue line is the distribution of the original data. br023_new, alcohol intake; depsoc_new, social deprivation; isced1997_r, educational attainment; iadl, instrumental activities of daily living; bmi, body mass index. m=40*
